# College and Hospital Notes

**Published:** 1911-03

**Authors:** 


### COLLEGE AND HOSPITAL NOTES

---

The new isolated pavilion of the Children's Hospital of Buffalo for the care of contagious cases has been completed, and is ready for the reception of patients. Twenty-five children can be accommodated in the several public and private wards. The pavilion has its own operating room and every precaution against the spreading of infection has been taken.

---

After much discussion and the display of considerable feeling on the part of residents in various parts of Buffalo, where it was proposed to locate a new city hospital, a site has been finally selected which meets not only with the approval of the various committees having interest in the project but is welcomed by the residents of the neighborhood. The site is known as the West farm and is located at the south west corner of Kensington Avenue and Grider Street, Buffalo. It has a frontage of 2,800 feet on Grider street and 1,500 on Kensington Avenue. The site is readily accessible by street car lines and contains 81 acres. The reported price is $225,000. The location is approximately the highest point in the city.

---

A campaign has been inaugurated with a view to raising $200,000 for the purpose of erecting a new hospital for the German Deaconess Hospital Society of Buffalo, adjacent to the present building on Kingsley street. Henry W. Simon, Dr. Julius Richter, Oscar F. Georgi, W. G. Bischop, H. F. Rielke and P. H. Schalbacher compose the executive committee which will be in charge of the campaign. There are about 40 churches affiliated with the German Deaconess work in Buffalo. The proposed building is to be of fireproof construction 46 feet wide and 196 deep with four stories and basement; construction will be of white brick and the trimmings are to be of white cut stone.

---

THE Ernest Wende Hospital, at Broadway and Spring street, Buffalo, the city institution which is provided for the care of contagious cases has been completely rebuilt at a cost of about $25,000 and announcement is made by Dr. W. S. Goodale, superintendent, that there is ample provision for the care of as many cases of scarlet fever, diphtheria and erysipelas as may arise under normal conditions.

---

MONTGOMERY county has decided to build a tuberculosis hospital under the provisions of the Hamilton-Whitney law passed by the New York state legislature last year. Ten other counties are preparing to establish similar hospitals.

